# A Java Program for LRE-Based Real-Time qPCR that Enables Large-Scale Absolute Quantification

**DOI:** 10.1371/journal.pone.0017636

**Published:** 2011-03-02

**Authors:** Robert G. Rutledge

**Affiliations:** Natural Resources Canada, Canadian Forest Service, Quebec, Canada; Texas A&M University, United States of America

## Abstract

**Background:**

Linear regression of efficiency (LRE) introduced a new paradigm for real-time qPCR that enables large-scale absolute quantification by eliminating the need for standard curves. Developed through the application of sigmoidal mathematics to SYBR Green I-based assays, target quantity is derived directly from fluorescence readings within the central region of an amplification profile. However, a major challenge of implementing LRE quantification is the labor intensive nature of the analysis.

**Findings:**

Utilizing the extensive resources that are available for developing Java-based software, the LRE Analyzer was written using the NetBeans IDE, and is built on top of the modular architecture and windowing system provided by the NetBeans Platform. This fully featured desktop application determines the number of target molecules within a sample with little or no intervention by the user, in addition to providing extensive database capabilities. MS Excel is used to import data, allowing LRE quantification to be conducted with any real-time PCR instrument that provides access to the raw fluorescence readings. An extensive help set also provides an in-depth introduction to LRE, in addition to guidelines on how to implement LRE quantification.

**Conclusions:**

The LRE Analyzer provides the automated analysis and data storage capabilities required by large-scale qPCR projects wanting to exploit the many advantages of absolute quantification. Foremost is the universal perspective afforded by absolute quantification, which among other attributes, provides the ability to directly compare quantitative data produced by different assays and/or instruments. Furthermore, absolute quantification has important implications for gene expression profiling in that it provides the foundation for comparing transcript quantities produced by any gene with any other gene, within and between samples.

## Introduction

Real-time qPCR has provided the foundation for a plethora of applications in basic research, biomedical diagnostics and pathogen detection [1], [2], [3]. Nevertheless, the relative quantification upon which conventional qPCR methodologies are based has prevented the full potential of real-time qPCR from being realized. Foremost is the difficulty of implementing absolute quantification, due to the necessity of constructing target-specific standard curves [4]. This makes absolute quantification impractical for large-scale applications that require quantification of more than a handful of targets.

Originating from the application of sigmoidal mathematics to model PCR amplification, linear regression of efficiency (LRE) provides an alternative approach to real-time qPCR, in which absolute quantification can be conducted without standard curves [5], [6], [7]. In addition to enabling large-scale absolute quantification, LRE provides quality control capabilities not possible with conventional methods. Finally, extensive testing has demonstrated the ability to achieve absolute accuracies of ±15–30%, even down to a single target molecule [7].

Despite the exceptional capabilities of LRE, attempts to manually implement data analysis using MS Excel quickly became untenable. This in turn prompted attempts to develop software for automated analysis, which led to the production of a small Java program that automated LRE quantification [5]. Unfortunately, this program was limited to analysis of one amplification profile at a time, and provided no ability to store data. Taking advantage of the extensive resources that are freely available for developing Java-based software, it was possible to extend this simple Java program into a fully featured desktop application. Called the LRE Analyzer, this program provides the automated data analysis and database capabilities required for implementing large-scale qPCR applications.

## Methods

### Implementation

The LRE Analyzer was written in Java using the NetBeans IDE (http://netbeans.org), utilizing the modular architecture and windowing system provided by the NetBeans Platform. The object database DB4O (http://db4o.com) is used for data storage and JExcel (http://jexcelapi.sourceforge.net) is used for data import and export. The program source code and installation files have been published as an open source project at Google Code (http://code.google.com/p/lreqpcr) under a GNU GPL license, in addition to a website that provides supporting information (https://sites.google.com/site/lreqpcr). The program has been tested extensively using the MS Windows XP operating system and has been confirmed to run on the Mac OS X and Unix operating systems (with JRE 1.6 installed).

### Installation

The LRE Analyzer can be installed by downloading the files provided at the open source project website (http://code.google.com/p/lreqpcr), which also includes demonstration database files.

### cDNA quantifications

RNA extraction and reverse transcription were conducted as previously described [5], [7]. Data presented in the demonstration databases were generated using an Applied Biosystems 7500 instrument (normal ramping), Qiagen QuantiTect in a 10 µl reaction volume containing 500 nM of primers, in 96 well white plates (ABgene) sealed with MicroAmp film (Applied Biosystems), and amplified using a cycling regime of 15 min activation at 95°C, followed by 50 cycles of 95°C −10 s, 65°C −120 s. Limiting dilution assays (LDA) were conduced as previously described [5], [7].

## Results

### Databases and data structures

**Figure 1:**
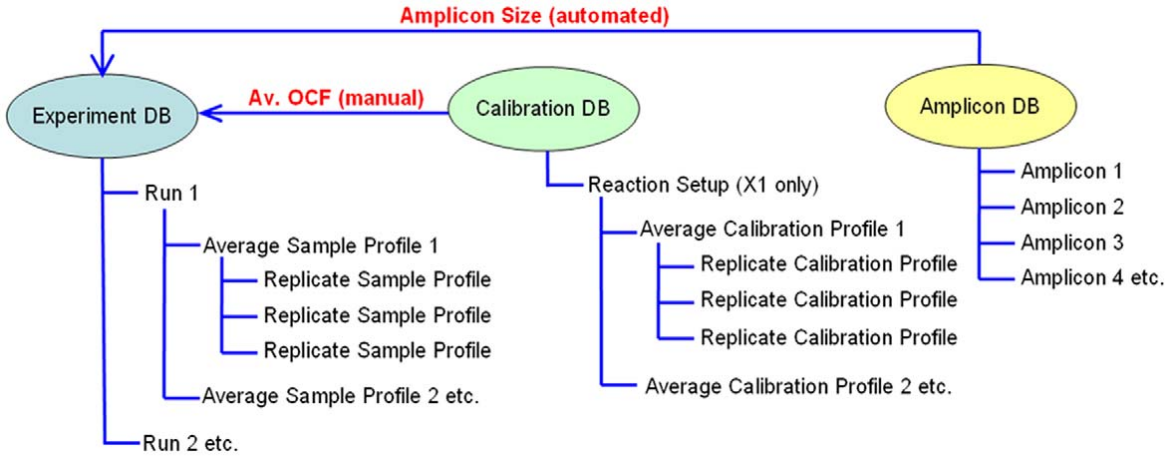
The three LRE Analyzer databases. The experiment database holds a group of related runs, a concept taken from the RDML guidelines for exchange of qPCR data [8]. Similarly, the calibration database holds calibration profiles used for optical calibration, whereas the amplicon database contains amplicon information. Data is organized into tree-like structures, which provides a convenient method for viewing and editing data. For the experiment database, each run is presented as a branch under which the profiles generated during each run are listed. As described in the text, the replicate sample profiles are used to generate an average sample profile. Similarly, the calibration database holds calibration profiles organized under a reaction setup, from which an average optical calibration profile (OCF) is generated. The primary function of the amplicon database is to provide amplicon sizes during run import, which in combination with an average OCF is used to determine the number of target molecules within each sample.

The LRE Analyzer stores data in three independent databases, maintained as files with distinct extensions: *.exp, *.cal and *.amp respectively (Figure 1). An important extension of LRE over conventional qPCR methods is construction of an average profile from their respective replicate profiles (i.e. technical replicates), generated by averaging, for each cycle, the fluorescence readings from each of the replicate profiles. This can greatly increase the precision of the fluorescence readings, which is essential for some instruments. Note that average profiles are automatically constructed during data import and are the primary working unit of the LRE Analyzer. However, an important qualification is that the replicate profiles must be tightly clustered. A prominent exception to this requirement occurs when target quantities fall below 10 molecules per reaction, due to the impact of Poisson distribution [7]. In such situations, target quantity must be determined by averaging the quantities produced by each individual replicate profile.

Another concept central to LRE quantification is referred to as “reaction setup”, which encompasses all of the factors impacting the optics of an assay. These include the reaction vessel and closure, the enzyme formulation and the optical characteristics of the instrument, which as a whole determine the fluorescence intensity of an assay. A key aspect of implementing absolute quantification is optical calibration in which the fluorescence intensity of an assay is quantified by amplification of a known quantity of lambda gDNA, generating what is called an optical calibration factor (OCF) that is unique to a specific reaction setup [5], [7].

Note that the LRE Analyzer help set provides detailed descriptions of how LRE quantification is conducted, along with guidelines on how to implement LRE-based assays. Demonstration database files are also provided to assist in illustrating how the LRE Analyzer functions, along with insights into the capabilities of LRE quantification.

### User Interface

**Figure 2:**
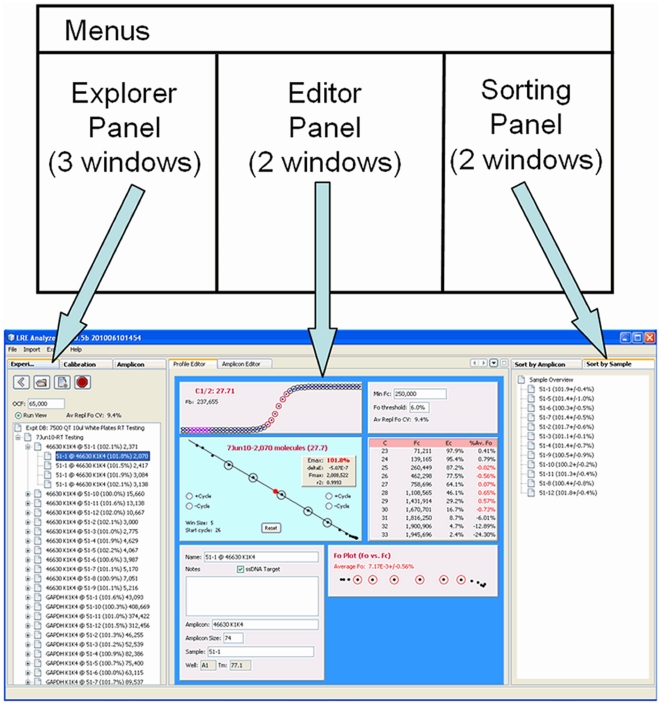
The user interface is organized into three panels. The explorer panel contains windows for viewing data within each of the three LRE database types (Figure 1). The central editor panel contains windows for viewing/editing of profiles and amplicons. The sorting panel allows viewing of profiles generated over multiple runs, sorted by either amplicon or sample. Note that the two sorting windows can be docked onto the left hand border of the main window, allowing the main window to be resized. This provides a convenient method for accessing the sorting windows while reducing the overall size of the main window. Note also that changes to the main window are saved across sessions so that the main window is restored to its previous state when the program is restarted.

**Figure 3:**
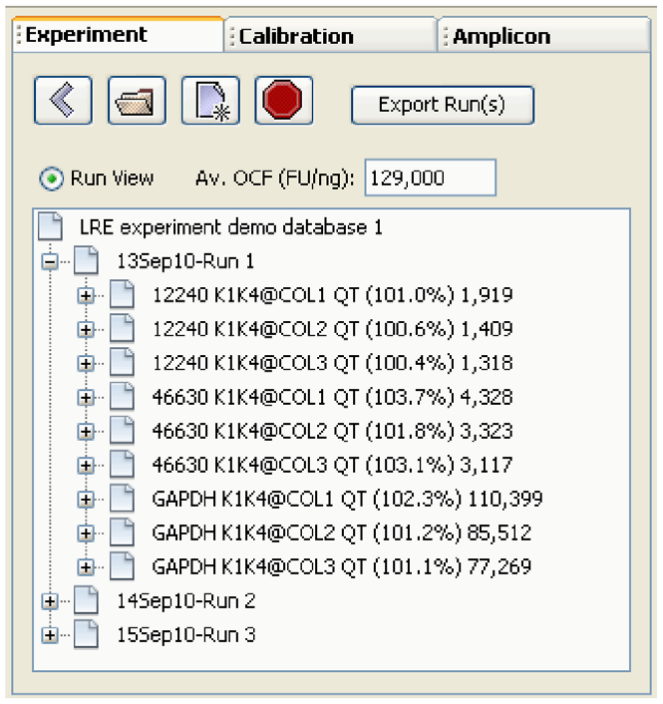
The explorer panel contains three windows for viewing of data within the three LRE databases. In this screenshot, the demonstration experiment database has been opened, which contains data generated by three runs conducted over 3 days, with the first run expanded to show nine average sample profiles. The sample profile labels are generated using the template: <“amplicon name” @ ”sample name” (E_max_) # target molecules>, and illustrate the primary output of the LRE Analyzer, which is the automated determination of the number of target molecules within a sample. In this example, transcript quantities have been determined for three genes (12240, 46630 and GAPDH) within three replicate reverse transcriptase reactions (COL1-3).

**Figure 4:**
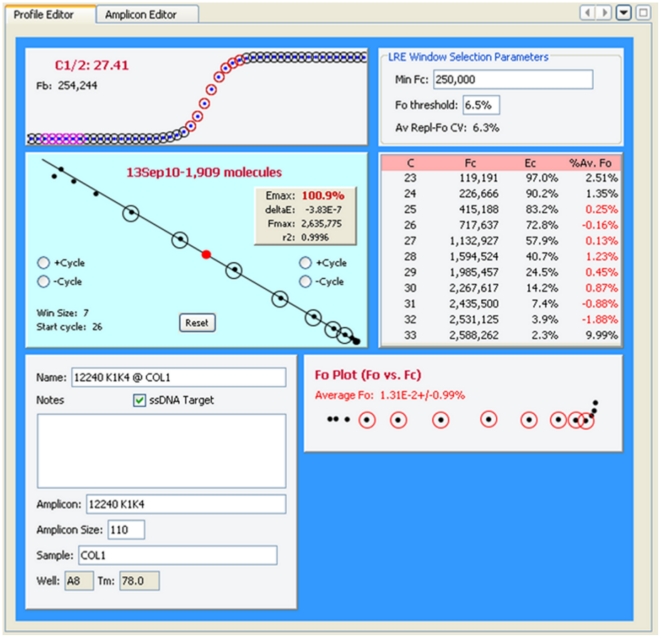
The editing panel contains windows for editing profiles and amplicons. In this example, a replicate sample profile has been selected in the experiment database explorer window, which triggers a display of information associated with this profile. Although a description of each of the subpanels will not be presented here, the LRE Analyzer help set provides a detailed description of how each one functions, along with how the number of target molecules is determined, which in this example is 1,909 molecules.

**Figure 5:**
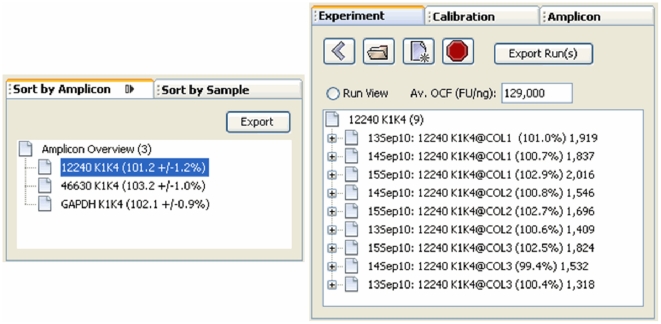
The sorting panel allows profiles to be organized by either amplicon or sample. These windows become active whenever the experiment or calibration explorer window is active. Selection of either an amplicon or sample (an amplicon in this example) will generate a list of all profiles within the database (an experiment database in this example) generated with that amplicon or sample. This in turn allows profiles generated across multiple runs to be viewed/edited or exported as a group. Note that selecting the “Run View” button within the experiment explorer window will restore the run-based tree view. Note also that the sorting windows can be iconized using the button located in the upper right side of each window. Placing the mouse over either of the iconized windows will trigger the respective window to fly out, allowing an item to be selected. Selecting an item in the explorer window will then trigger retraction of the sorting window, providing a convenient method for accessing the sorting windows while reducing the overall size of the main window.

The user interface is organized into three panels, each containing windows that provide functions for viewing and/or editing of data from each of the three LRE database types (Figure 2). The explorer panel contains windows that allow creation, opening and closing of database files. Once a database file is opened, data is presented as a tree, such as profiles within a run, along with displaying information about the data element within its label (Figure 3). When a data element within an explorer window is selected, the corresponding editor window appears within the editor panel, displaying information about the selected data element, much of which can be edited (Figure 4). The sorting panel makes it possible for profiles to be organized by either amplicon or sample, allowing profiles generated across multiple runs to be viewed/edited or exported as a group (Figure 5).

### Workflow

**Figure 6:**
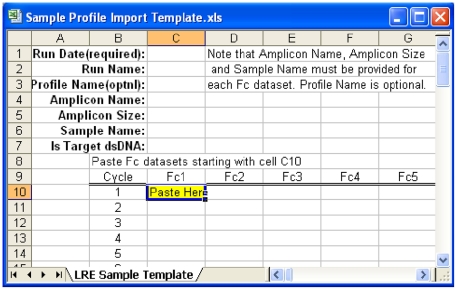
An Excel template provided by the LRE Analyzer for manual import of sample profiles. A similar import template is provided for calibration profiles. Raw fluorescence readings for each replicate profile are pasted into the template, along with amplicon and sample name, amplicon size and the strandedness of the target, for each replicate profile. The Excel workbook is then selected within the LRE Analyzer to initiate profile import. Note that details about data import and export are provided within the LRE Analyzer help set.

**Figure 7:**
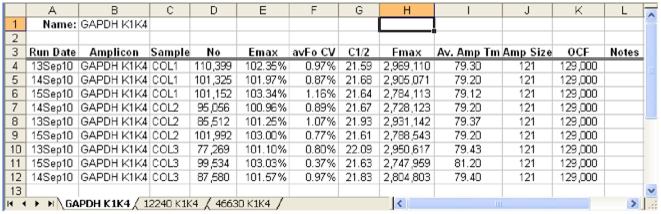
An example of data export sorted by amplicon, in which profiles generated by each amplicon are placed into a separate worksheet. Data sorted by run or sample are similarly exported. No: the number of target molecules; C1/2: the fractional cycle at which reaction fluorescence reaches half of maximum (similar to, but more reliable than, C_q_).

Following completion of a run, fluorescence readings are exported into an Excel workbook. The LRE Analyzer provides Excel templates for manual data import (Figure 6), along with support for importing data from Applied Biosystems 7500 and Stratagene Mx3000p instruments (support for additional platforms could be added depending on demand and available resources). The primary requirement for data import is to link each amplification profile to the sample and amplicon used to generate it, and to designate which profiles are calibration profiles. The data is then read into the LRE Analyzer, which conducts automated analysis for each profile, including automated retrieval of amplicon size from an amplicon database. Although it is possible to manually adjust the analysis by adjusting the LRE window, testing has shown that automated analysis can routinely generate quantitative accuracies in the ±15–30% range. Target quantities can then be exported into an Excel workbook sorted by run, sample or amplicon (Figure 7).

### Automated LRE window selection

#### Implementing a simple strategy

**Figure 8:**
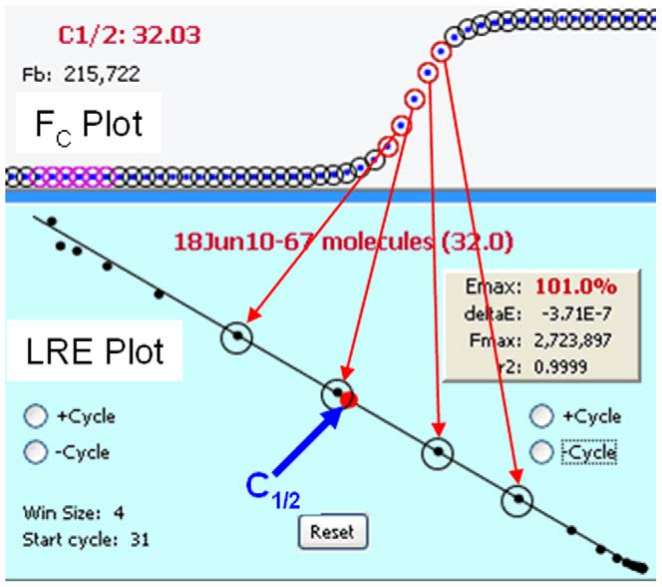
Defining the LRE window. The LRE window encompasses a contiguous group of cycles that are used for LRE analysis of a profile, depicted as red and black circles within the F_C_ and LRE plots, respectively. Linear regression analysis is then used to determine values for the two parameters upon which LRE analysis is based (E_max_ from the Y-intercept and ΔE from the slope). Although LRE window selection is fully automated, the program does allow manual adjustment of the LRE window using the buttons within the LRE plot.

In addition to conducting the mathematical calculations required for LRE quantification [5], [7], devising a method for automated LRE window selection was essential for the general efficacy and reliability of the LRE Analyzer. As described in Figure 8, the LRE window consists of the cycles that are included in the LRE analysis of a profile. The basic strategy was to select the first cycle of the LRE window (referred to as the “start cycle”), which defines the bottom of the LRE window, and to start with a default window size of three cycles. The LRE window is then expanded by adding cycles to the top of the window, until a cycle is encountered that exceeds a specified threshold based on conformity to the LRE model.

#### Optical read precision is critical for start cycle selection

Although a primary objective is to maximize the size of the LRE window by placing the start cycle as early in a profile as possible, optical read precision becomes a major limitation. This is because LRE analysis is based on determining cycle efficiency (E_C_), which is calculated by dividing the cycle fluorescence (F_C_) by the fluorescence reading produced by the preceding cycle (F_C_-1):
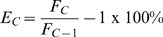



Due to this ratio-based determination, the accuracy of E_C_ determination is dependent on the precision of the fluorescence readings, which is generally referred to as “read precision”. The accuracy of cycle efficiency determination can thus be dramatically compromised when reaction fluorescence is below the lower limit of the instrument's optical capacity. Large differences in this lower limit between different instruments, combined with the arbitrary nature of the fluorescence units used in real-time PCR, presented major challenges for automated selection of the start cycle.

**Figure 9:**
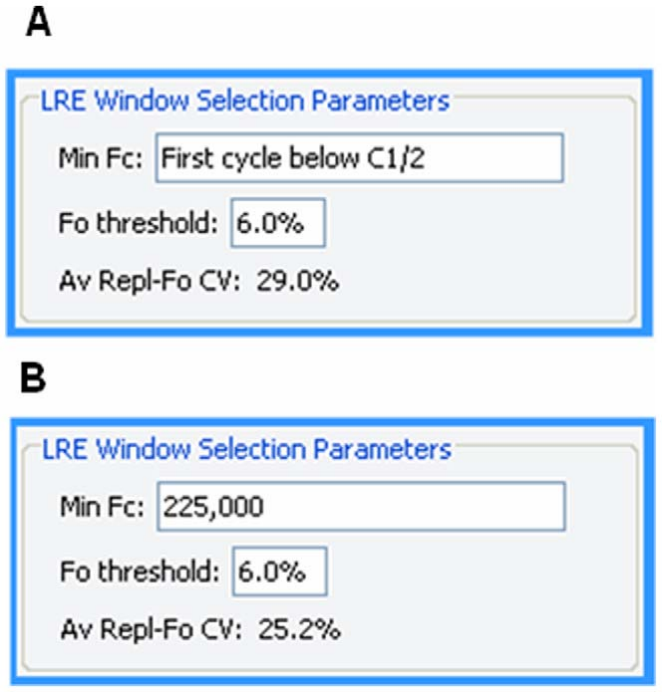
The LRE window selection parameters panel. **A**. Default settings in which the start cycle is set to the first cycle below C_1/2_ and the F_0_ threshold set to 6.0%, which is used to determine the top of the LRE window (see the text for details). This produces an average replicate F_0_ CV (Av Repl-Fo CV) of 29.0%, which is a general indicator of intra-run variance generated by LRE analysis (see the text for details). **B**. The minimum F_C_ has been manually set to 225,000 fluorescence units, such that the start cycle is set to the cycle following the first cycle that generates a fluorescence reading above this minimum F_C_. This reduces the average replicate F_0_ CV to 25.2%. Note that the LRE Analyzer help set provides additional details about LRE window selection.

This prompted a default implementation based on a simple, albeit suboptimal method of designating the start cycle as the first cycle below C_1/2_. However, although this approach can be reasonably reliable using a number of reaction setups, an alternative method was developed that allows the lower limit of the LRE window to be manually specified. Based on entry of a “minimum F_C_”, the start cycle is set to the cycle following the first cycle that produces a F_C_ greater than this minimum (i.e. the cycle from which the start cycle E_C_ denominator is taken). The “LRE Window Selection Parameters” panel within the Profile Editor window allows the minimal F_C_ and F_0_ threshold to be adjusted manually (Figure 9).

During early implementation of the LRE window selection parameters, it became apparent that a method for assessing the overall quantitative precision could be useful. The approach taken was based on the variance of target quantities generated by technical replicates; that is, the CV of the F_0_ values produced by replicate profiles. Referred to as the “Av Repl-Fo CV”, averaging the quantitative variances generated by all the replicate reaction sets within a run, provided such a general assessment. This not only proved to be useful for selecting an optimal minimum F_C_, but also for assessing the overall performance of an assay. Although beyond the scope of this study, this has revealed, among other things, large differences in instrument performance, due primarily to differences in the optical precision they produce. A simple but generally effective method is to lower the minimum F_C_ until the average replicate F_0_ CV reaches a minimum, although this should only be taken as a general guideline, as exceptions have been encountered.

#### Defining the top of the LRE window via the F_0_ threshold

**Figure 10:**
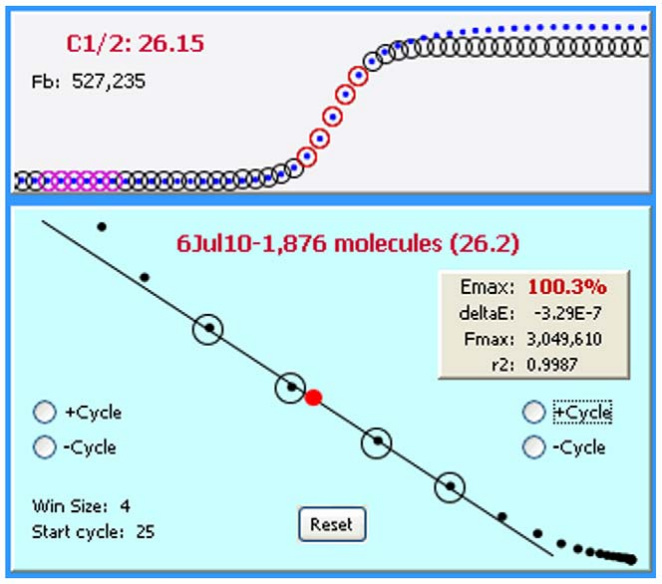
An example of plateau drift. A common form of kinetic distortion, referred to as “plateau drift”, is produced by a continued increase in amplicon DNA quantity beyond that predicted by the LRE model, which is represented as circles within the upper panel (referred to as the F_C_ plot). This is particularly evident in the LRE plot (lower panel) as a progressive drifting of points above the LRE line. Importantly, inclusion of these aberrant cycles in the LRE window (represented by the black circles in the LRE plot and the red circles in the F_C_ plot) will generate an underestimation of E_max_ (Y-intercept) that leads to an overestimation of target quantity.

A major source of quantitative error discovered during early attempts to apply sigmoidal mathematics to PCR using nonlinear regression analysis (sigmoidal curve fitting or SCF) were distortions within the upper region of a profile [9]. In order to maximize quantitative accuracy, it was found essential to exclude such aberrant cycles from the analysis. The recursive nature of LRE analysis provided a simple method for identifying such aberrant cycles [5], which are apparent in both the F_C_ and LRE plots (Figure 10).

**Figure 11:**
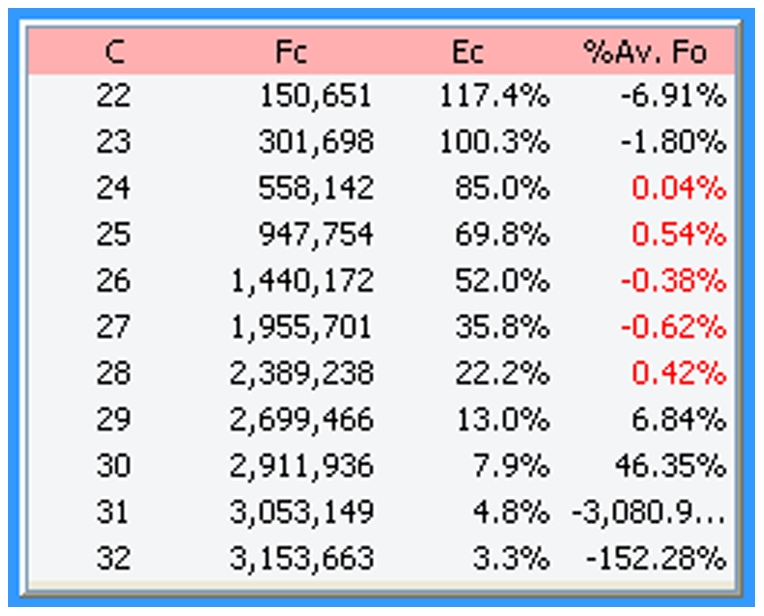
The tabular summary provides values for the three parameters that define a cycle. C: cycle number, F_C_: the fluorescence reading, E_C_: the cycle's amplification efficiency, %Av. Fo: the percent difference between the cycle F_0_ and the average F_0_ generated by the cycles within the LRE window (designated by the red font). In this example, the F_0_ threshold (Figure 9) was set to 6% so that cycle 29, which generated a 6.84% difference, triggered termination of LRE window expansion at cycle 28.

An important objective for setting the upper limit of the LRE window was thus to avoid inclusion of such aberrant cycles. An objective method came from taking the F_0_ value generated by the cycle immediately above the LRE window, and comparing it with the average F_0_ value generated by the cycles within the LRE window. If the difference is below a specified value, defined as the F_0_ threshold (Figure 9), the LRE window is expanded to include this next cycle and LRE analysis is repeated. This process is continued until a cycle is encountered that exceeds the F_0_ threshold. The Tabular Summary located within the profile editing panel provides a numerical perspective on the process (Figure 11).

**Figure 12:**
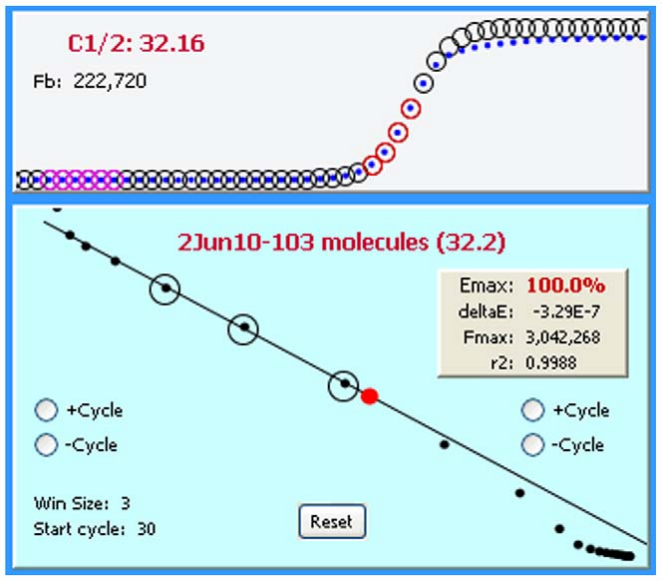
An example of profile collapse characterized by progressive drifting of points below the LRE line. In this example the collapse is produced by the primer pair, although reduced enzymatic activity, such as that produced at high concentrations of SYBR Green I, have also been found to generate profile collapse for primer pairs that normally conform well to the LRE model. Similar to plateau drift (Figure 10), it is important to exclude such aberrant cycles from the LRE window, which for profile collapse will lead to an overestimation of E_max_ (Y-intercept), which will generate an underestimation of target quantity.

Although an F_0_ threshold of 6% has generally been found to be effective, it should be noted that increasing the F_0_ threshold can lead to susceptibility to another form of kinetic distortion, referred to as “profile collapse” (Figure 12). In contrast to plateau drift that can generate underestimations of E_max_, inclusion of collapsed cycles overestimates E_max_, which in turn generates an underestimation of target quantity.

#### Another form of aberrant kinetics

**Figure 13:**
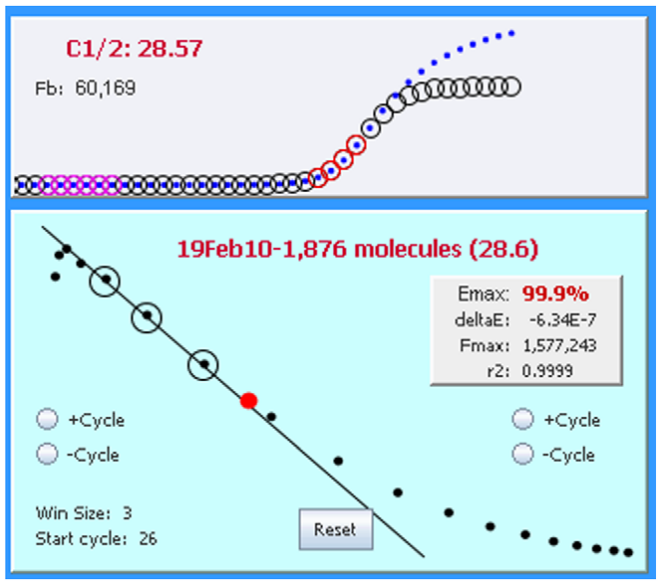
An example of extreme profile arcing produced by the enzyme formulation. Similar to plateau drifting (Figure 10), inclusion of these aberrant cycles into the LRE window will generate underestimations of E_max_ (Y-intercept).

Another form of kinetic distortion found to be produced by some commercial enzyme formulations, is referred to as “profile arcing” (Figure 13). Such profiles do not conform well to the LRE model and can thus generate unreliable quantifications. Enzyme formations that have been found to be effective for LRE analysis include Qiagen QuantiTect, Agilent Brilliant II, and Invitrogen Platinum SYBR, although many others are likely to also be effective. Finally, it should be noted that although extensive testing has shown SYBR Green I to be effective for LRE-based absolute quantification, other detection chemistries many not be as effective.

### An example taken from the demonstration databases

**Figure 14:**
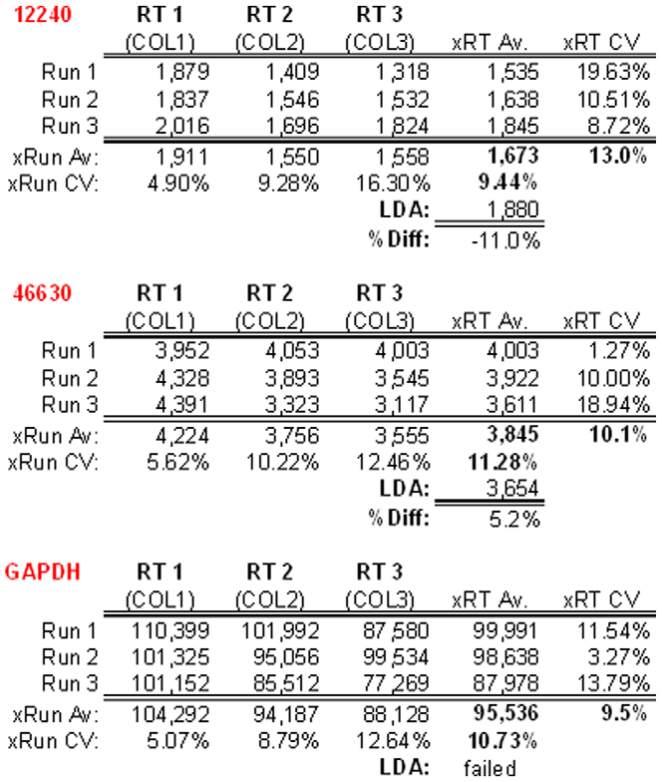
Excel summary of the cDNA quantifications contained within the demonstration experiment database. Expressed as the number of transcripts per 2.5 ng of total RNA, these datasets provide insights into both the utility of absolute quantification and the precision that can be generated by the LRE Analyzer. This reveals a run-to-run CV of ±5–16% for all three targets, and a RT-to-RT CV of ±1–20%. This summary also presents LDA quantifications that provide an independent determination of target quantity, with the difference expressed as a percentage (%Diff). Note that the GAPDH LDA failed due to the frequent production of non-specific products when the target quantity was diluted below one molecule per aliquot.

Generated during a study investigating the reproducibility of LRE quantification for gene expression profiling, the datasets provided in the demonstration databases focus on assessing the reproducibility of reverse transcription and run-to-run quantitative variances. Consisting of three runs conducted over a 3-day period, three Arabidopsis reference genes (12240, 46630 and GAPDH) were quantified within each of three replicate reverse transcriptase (RT) preparations (i.e. made with the same RNA sample) using three replicate PCR reactions for each quantitative determination. Figure 14 compares target quantities generated across both runs and RT preparations for each of the three targets that produced an overall run-to-run average CV of ±9.5% (i.e. across all three targets), which is substantially below that reported previously (±15–30%) [5], [7], likely due to the exceptional optical precision produced by the Applied Biosystems 7500 instrument used to conduct these runs.

Of potentially greater significance is that these datasets reveal RT variances of 13.0%, 10.1% and 9.5%, respectively, for each of three targets (average 10.9%). This indicates that these RT reactions generated variances below the variance of LRE quantification (i.e. error of measurement), which in turn demonstrates the high level of repeatability that can be achieved with reverse transcription. It is also important to note that these target quantities presented here were generated using the automated analysis provided by the LRE Analyzer, which requires little or no user intervention. Figure 14 also provides an example of a key attribute of absolute quantification, which is the ability to assess quantitative accuracy using limiting dilution assay (LDA), a method that provides the ability to conduct absolute quantification independent of the kinetic and optical parameters on which real-time qPCR is based [5], [7]. This indicated that for two of the targets, LRE quantification agreed within 12% of that generated by LDA, again consistent with that previously reported [5], [7].

Finally, the LRE Analyzer provides an open source platform that facilitates data storage and exchange. For example, the LRE databases could be published as supplementary data that in turn would allow access to raw data in an organized, easy to access form.

## Discussion

One of the most striking features of using the LRE Analyzer is the ease of evaluating large amounts of data generated over multiple runs. Central to this capability is the universal perspective provided by absolute quantification, which allows target quantities to be directly compared not only across different runs, but also across different assays and/or instruments. In contrast, the relative quantification upon which conventional qPCR methods rely, generates target quantities based on a single point that defines the position of a profile. Called the “quantitative cycle” (Cq) [10], a major limitation of this approach is that Cq is assay specific. Thus, in order to directly compare Cq values, some form of normalization is required, such as conducting run normalization using external standards [11], or normalization to a reference gene(s) [12].

Absolute quantification eliminates the need for such post-run data processing, in that absolute quantification normalizes assay-specific differences, such that quantities expressed as the number of target molecules become independent of assay implementation. An important implication of this principle is that assay performance can be defined in terms of quantitative accuracy; that is, how well a quantitative determination correlates with the actual number of target molecules within a sample [7].

The ease of conducting absolute quantification provided by LRE, combined with the automated data processing capability of the LRE Analyzer, could thus greatly enhance the utility and reliability of real-time qPCR. In addition to circumventing many of the limitations associated with conventional methods, the universal perspective provided by absolute quantification also provides the foundation for effective standardization of qPCR that, for example, could be achieved through the establishment of performance benchmarks based on quantitative accuracy [5], [7].
